# Development of a novel HAC-based “gain of signal” quantitative assay for measuring chromosome instability (CIN) in cancer cells

**DOI:** 10.18632/oncotarget.7854

**Published:** 2016-03-02

**Authors:** Jung-Hyun Kim, Hee-Sheung Lee, Nicholas C. O. Lee, Nikolay V. Goncharov, Vadim Kumeiko, Hiroshi Masumoto, William C. Earnshaw, Natalay Kouprina, Vladimir Larionov

**Affiliations:** ^1^ Developmental Therapeutics Branch, National Cancer Institute, NIH, Bethesda, MD, USA; ^2^ School of Biomedicine, Far Eastern Federal University, A. V. Zhirmunsky Institute of Marine Biology, FEB RAS, Vladivostok, Russia; ^3^ Laboratory of Cell Engineering, Department of Human Genome Research, Kazusa DNA Research Institute, Kisarazu, Japan; ^4^ Wellcome Trust Centre for Cell Biology, University of Edinburgh, Edinburgh, Scotland

**Keywords:** chromosome instability, CIN, human artificial chromosome, HAC, anticancer drugs

## Abstract

Accumulating data indicates that chromosome instability (CIN) common to cancer cells can be used as a target for cancer therapy. At present the rate of chromosome mis-segregation is quantified by laborious techniques such as coupling clonal cell analysis with karyotyping or fluorescence *in situ* hybridization (FISH). Recently, a novel assay was developed based on the loss of a non-essential human artificial chromosome (HAC) carrying a constitutively expressed EGFP transgene (“loss of signal” assay). Using this system, anticancer drugs can be easily ranked on by their effect on HAC loss. However, it is problematic to covert this “loss of signal” assay into a high-throughput screen to identify drugs and mutations that increase CIN levels. To address this point, we re-designed the HAC-based assay. In this new system, the HAC carries a constitutively expressed shRNA against the *EGFP* transgene integrated into human genome. Thus, cells that inherit the HAC display no green fluorescence, while cells lacking the HAC do. We verified the accuracy of this “gain of signal” assay by measuring the level of CIN induced by known antimitotic drugs and added to the list of previously ranked CIN inducing compounds, two newly characterized inhibitors of the centromere-associated protein CENP-E, PF-2771 and GSK923295 that exhibit the highest effect on chromosome instability measured to date. The “gain of signal” assay was also sensitive enough to detect increase of CIN after siRNA depletion of known genes controlling mitotic progression through distinct mechanisms. Hence this assay can be utilized in future experiments to uncover novel human CIN genes, which will provide novel insight into the pathogenesis of cancer. Also described is the possible conversion of this new assay into a high-throughput screen using a fluorescence microplate reader to characterize chemical libraries and identify new conditions that modulate CIN level.

## INTRODUCTION

Abnormal chromosome number (aneuploidy) is a known feature of most solid tumors and is often accompanied by an elevated rate of chromosome mis-segregation termed chromosome instability (CIN) [1]. The gain or loss of entire chromosomes leads to large-scale changes in gene copy number and expression levels. The molecular mechanisms underlying CIN include defects in chromosome cohesion, mitotic checkpoint function, kinetochore-microtubule attachment dynamics, cell cycle regulation as well as defects in DNA replication. Mutations in *CIN* genes are thought to be an early event in tumor development, predisposing cells to the accumulation of genetic changes leading to progression to a cancerous state [2-4]. Notably, a significant fraction of human *CIN* genes remains unidentified and in part this is due to the lack of a simple assay to detect CIN in vertebrate cells, similar to that developed for yeast cells [5]. Thus, there is an important need to develop improved assays for measuring chromosome transmission fidelity in human cells.

While CIN can drive cancer genome evolution and tumor progression, recent findings point to the existence of a threshold level beyond which CIN becomes a barrier to tumor growth. Therefore excessive CIN can be exploited therapeutically [6-11] and evaluation of CIN as an approach to cancer therapy is an attractive strategy. However, drugs known to increase CIN beyond the therapeutic threshold are currently few in number. Hence, a screen of established anticancer drugs as well as novel drugs to rank their CIN potency is warranted.

Typically, rates of chromosome mis-segregation have been quantified by laborious techniques such as coupling clonal cell analysis with karyotyping or fluorescence *in situ* hybridization (FISH) [12-14] In our recent work, we developed a quantitative assay for measuring CIN [15] that is based on the use of a non-essential human artificial chromosome (HAC) with a functional kinetochore [16-20]. Specifically we used a HAC constructed for gene delivery that contains a single gene-loading site [21-23]. To adapt this HAC for CIN studies, a constitutively expressed *EGFP* transgene was inserted into it [15]. Cells that inherit the HAC display green fluorescence, while cells lacking the HAC do not (“loss of signal” assay). This allows the measurement of HAC loss rate by routine flow cytometry (details of this assay are shown in Supplementary Figure S1).

There are several advantages of the HAC-based assay compared to karyotype analysis or micronucleus tests that are commonly used to study CIN and its induction by environmental agents. First, the HAC-based assay is significantly faster and less labor intensive. Second, the flow cytometer can readily analyze tens of thousands of cells compared to the hundred or so cells the latter two methods can analyze. Thus, the measurements are more precise. Finally, while the HAC contains a functional centromere/kinetochore and is efficiently transferred at mitosis, its relatively small size (∼1 Mb) [24] causes a frequency of spontaneous HAC loss roughly 10-fold higher than that of native chromosomes [15, 16], making the HAC a sensitized model for measuring CIN. Together, these features of the HAC allow detection of small differences between frequencies of chromosome loss induced by different compounds. This is important because accurate assessment of CIN is crucial to select drugs with the highest effect on chromosome transmission. In our recent study, the EGFP-HAC-based CIN assay was applied for analysis of 62 anticancer drugs corresponding to six groups of compounds with different mechanisms of action [25]. Within each group, drugs could be ranked with regards to their effect on the rate of HAC loss [25].

While EGFP-HAC offers sensitive, precise and simple means to measure CIN, development of a fast high-throughput screening method, based on the detection of fluorescence signal loss is problematic. This is mostly due to the difficulty to detect single cells with a decreased EGFP signal among the large number of the EGFP-positive cells during the first days after drug treatment. Herein, we developed a complementary HAC-based system, based on the gain of the EGFP fluorescence signal after HAC loss. In this system, the HAC carries a constitutively expressed shRNA against an *EGFP* transgene integrated elsewhere into a natural human chromosome. Thus, cells that inherit the HAC display no green fluorescence, while cells lacking the HAC do. The high precision of this new assay was verified by measuring HAC loss after cell treatment by several anticancer drugs corresponding to three groups of compounds with different mechanisms of CIN induction. In addition, both HAC-based assays have been proven to be applicable for screening human genes to identify phenotypes associated with CIN and thus to identify *CIN* genes themselves.

## RESULTS

### Experimental design for quantitative CIN measurement by “gain of signal” assay

**Figure 1:**
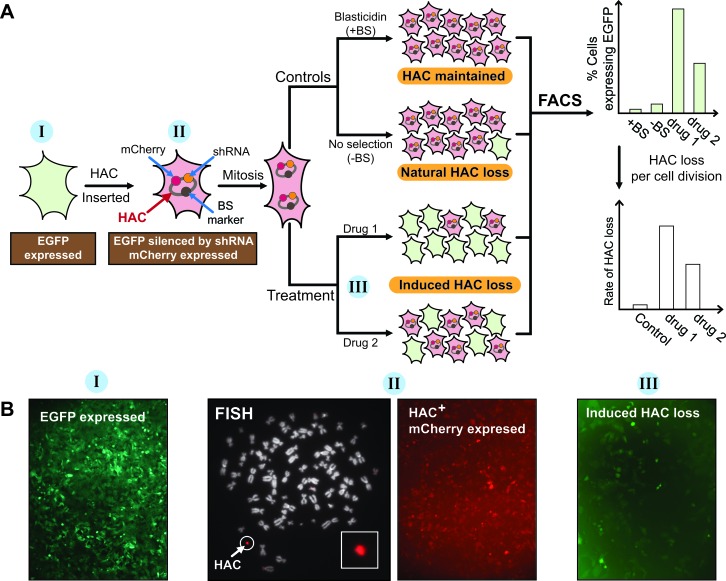
“Gain of signal” HAC-based assay to measure CIN level in cancer cells **A.** A general scheme of assay to measure chromosome instability (CIN), based on the HAC expressing a small hairpin RNA (shRNA) against the *EGF****P*** transgene integrated into a random chromosomal site of genome of the HT1080 human cell line. When EGFP is expressed it makes the cell green. Cells containing the mCherry-shRNA-HAC display a red signal. When cells loose the HAC, they become green again. Thus, it is expected that the control population of untreated cells show uniform red fluorescence while cell population that have lost HAC after drug treatment should show green fluorescence. The percentage of cells with EGFP expressed can be measured by FACS. Thus, the compounds, which increase HAC loss and, therefore, increase spontaneous chromosome mis-segregation rates, may be identified. **B.** Fluorescence images of human HT1080 cells expressing the *EGFP* transgene (exhibiting a green fluorescent signal) (I); the cells after transfer into them the mCherry-shRNA-HAC (exhibiting a red fluorescent signal) (II) and the same cells that have lost the HAC after drug treatment (exhibiting a green fluorescent signal) (III). FISH analysis of the HAC-containing HT1080 clone is also shown. The HAC was visualized using the BAC32-2-mer(tetO) DNA probe (red) (see for details MATERIALS AND METHODS).

Figure 1A shows the general scheme of the “gain of signal” assay developed for measuring chromosome instability (CIN) using a genetically marked human artificial chromosome (HAC). The *EGFP* (enhanced green fluorescence protein) transgene is integrated into a random chromosomal site of human HT1080 cells and is expressed, making the cells green (I) (Figure 1A and 1B). The HAC developed in hamster host CHO cells carries the *mCherryFP* (mCherry fluorescence protein) transgene and a constitutively expressed short hairpin RNA (shRNA) against *EGFP*. After transfer of the mCherry-shRNA-HAC into HT1080-EGFP-expressing cells, the HAC is maintained as a non-essential 47^th^ chromosome (II) due to the presence of its functional kinetochore and the cells maintaining the HAC become red (II) (Figure 1A and 1B). Normally, after growing in non-selective medium (−BS) (i.e. in the absence of selection for HAC), the majority of cells still retain the HAC (natural HAC loss). However, if proper chromosome transmission is impaired, the fraction of cells that lost the HAC is significantly increased. Thus, while the control untreated cells do not display green fluorescence the cells that have lost HAC after drug treatment (induced HAC loss) should exhibit green fluorescence (III) that can be detected by fluorescence microscopy or flow cytometry (Figure 1A and 1B). In principle, this new assay may provide a convenient and sensitive way to monitor CIN in human cells.

### Construction of a cell line for CIN phenotype screening

**Figure 2:**
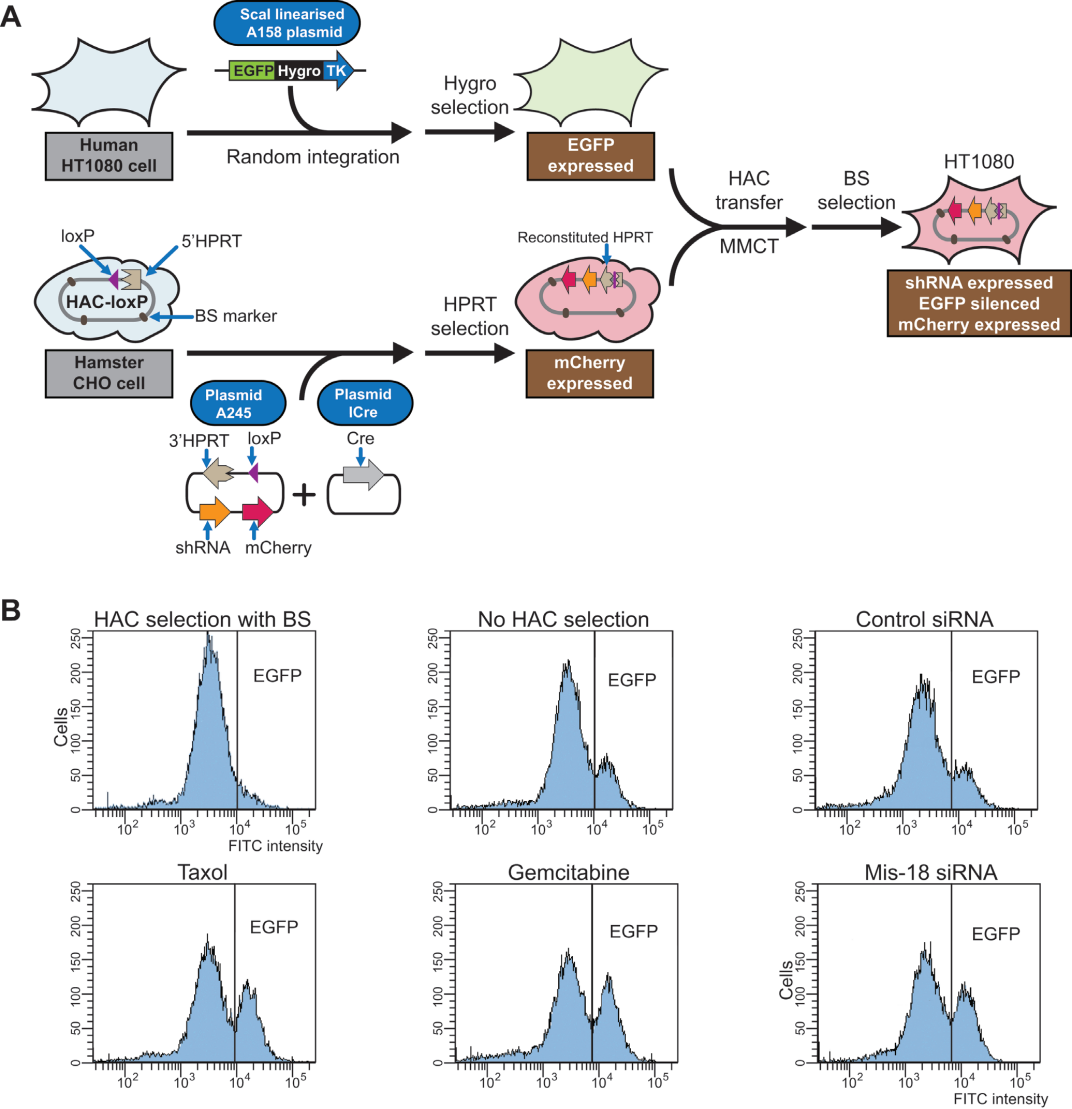
Development of “gain of signal” assay and its actual application for drugs screening **A.** General steps of development of “gain of signal” HAC-based assay. First, the *EGFP* transgene (plasmid A158) was integrated into a random chromosome site in human HT1080 cells, providing a stable expression of the EGFP protein (cells are green). Separately, the tDNA-shEGFP-mCherry plasmid (A245) was inserted into a unique gene-loading loxP site of alphoid^tetO^-HAC propagated in hamster CHO cells (cells are red). Then alphoid^tetO^-HAC carrying a constitutively expressed shRNA against *EGFP* was transferred into human HT1080 cells with the integrated *EGFP* transgene. After HAC transfer and shRNA expressed, the *EGFP* transgene is silenced and the cells become red. **B.** Flow cytometry histograms illustrating EGFP fluorescence before and after drug treatment. The x-axis represents the intensity of the fluorescence, the y-axis the number of cells. The results of triplicate experiments are shown.

To adapt the alphoid^tetO^-HAC [16] for CIN studies, the tDNA-shEGFP-mCherry plasmid (A245) (Supplementary Figure S2) was inserted into a single loxP loading site of the HAC [22] in HPRT-deficient Chinese hamster ovary (CHO) cells (Figure 2A). In the A245 construct, the mCherry-shRNA cassette is flanked by *tDNA* insulator sequences to protect the transgene from epigenetic silencing [26, 27]. Targeting of the cassette into the loxP site was accompanied by reconstitution of the *HPRT* gene, allowing cell selection on HAT medium. PCR analysis with specific primers (Supplementary Table S1) confirmed that the *HPRT* gene was indeed reconstituted in five randomly chosen drug-resistant clones. Based on fluorescence microscopy, all clones expressed mCherry. FISH analysis of CHO metaphase spreads revealed the HAC in an autonomous form in three analyzed clones (Supplementary Figure S3). One clone was chosen for further experiments.

The mCherry-shRNA-HAC was transferred from hamster CHO to the human HT1080 cells in which the *EGFP* transgene (plasmid A158) (Supplementary Figure S4) was integrated into a random chromosome site providing stable expression of the EGFP protein (clone HT1080-JH1) (Figure 2A). The HAC transfer was performed *via* microcell-mediated chromosome transfer (MMCT). Recipient cells were selected using the *BS* resistance gene on the alphoid^tetO^-HAC [16]. Three BS-resistant clones were isolated from one MMCT experiment and all three exhibited barely detectable expression of *EGFP,* indicating transgene silencing induced by the shRNA. FISH analysis showed that the HAC was maintained autonomously without detectable integration into the host genome in two out of three selected clones (Supplementary Figure S5A). One clone containing the mCherry-shRNA-HAC (clone HT1080-shGFP-4) was chosen for further analysis. In this clone, EGFP silencing was stable for at least six months under selective conditions (i.e. under selection for BS). Rare events of spontaneous HAC loss were accompanied by visual expression of EGFP. Based on these results, we conclude that the mCherry-shRNA is stably expressed from the HAC and that these cells may be exploited as a sensitized system for analyzing chromosome stability.

### Quantitative profiling of CIN induced through treatment by anticancer agents

We next investigated whether the mCherry-shRNA-HAC-based assay could be used to quantify the effects of compounds that cause chromosome loss or mis-segregation. For this purpose, we first chose seven anticancer drugs that have been previously ranked on their effects on HAC loss in HT1080 cells using the original “loss of signal” EGFP-HAC-based system (Supplementary Figure S1) [15, 25]. The cells with the autonomously propagated mCherry-shRNA-HAC were treated with two known aneugens: taxol (paclitaxel) and nocodazole, two HDAC inhibitors: romidepsin and SAHA, the poly(ADP-ribose) polymerase (PARP) inhibitor: olaparib, and the topoisomerase I inhibitor: LMP400.

For each compound, a cell cytotoxicity assay was carried out to determine IC50 values, i.e., the conditions under which the viability of cells would be around 50%. This parameter was chosen in order to normalize the results at the same percentage of viable cells [15]. Time treatments and drug concentrations corresponding to the IC50 are summarized in Table 1. After treatment, the cells were grown for two weeks in the absence of selection. Samples were analyzed every few days, and the proportion of fluorescent cells was determined by FACS. Green-fluorescent cells could be detected after a few days, and treated and untreated cell populations were clearly distinguishable after 5-7 days. The delay between HAC loss and the appearance of non-fluorescent cells is due to the persistence of the shRNA against EGFP. The sampling time had a broad interval (5-14 days) without a significant affect on the calculated rate of HAC loss similar to that previously determined for the original “loss of signal” system [15, 25]. Figure 2B shows representative flow cytometry histograms illustrating a gain of green fluorescence in cell populations treated by a single doze of taxol or gemcitabine. Importantly, the measurements were highly reproducible: the raw FACS data of three independent populations for drug treatments have standard deviations less than 1%.

**Table 1:** List of compounds used in this study

Drugs*	Target	IC50**
Taxol	Microtubule Stabilizing	10 nM
Nocadazole	Microtubule Destabilizing	5 μM
SAHA	Histone Deacetylase	2 μM
Romidepsin	Histone Deacetylase	2 nM
Olaparib	Poly ADP Ribose Polymerase	10 μM
LMP400	Topoisomerase I	50 nM
Gemcitabine	Ribonucleotide Reductase	50 nM
PF-2771	CENP-E	10 μM
GSK923295***	CENP-E	10 μM
Zoledronic acid	CENP-F	10 μM
**siRNA target**	Sense sequence as ordered****	**siRNA per well** **(6-well plate)**
SKA3	5′-AGACAAACAUGAACAUUAAUU-3′	25 pmol
CENP-E	5′-AACACGGAUGCUGGUGACCUC-3′	25 pmol
MIS18β	5′-AGGCAGUACUUACAACCUUUU-3′	25 pmol
ON-TAGET plus, Non-targeting pool	5′-UGGUUUACAUGUCGACUAA-3′ 5′-UGGUUUACAUGUUGUGUGA-3′ 5′-UGGUUUACAUGUUUUCUGA-3′ 5′-UGGUUUACAUGUUUUCCUA-3′	25 pmol

* All drugs, except RF-1771, GSK92395 and zoledronic acid, were described in our previous publications [15, 25]. GSK92395 was purchased from Selleckchem (Catalog no. S7090). PF-1771 was kindly provided by Dr. B.W. Murray (Pfizer Worldwide Research and Development, La Jolla Laboratories, San Diego). Zoledronic acid was purchased from Novartis Pharma AG (Stein, Switzerland).

** The cells were treated overnight.

*** For HT1080 cells containing EGFP-HAC, IC50 for GSK923295 was 2 μM. For HT1080 cells containing mCherry-shRNA-HAC, IC50 was 10 μM.

**** The two underlined uracil (UU) were added by Dharmacon as part of siRNA construction.

FISH analysis was used to confirm that the appearance of fluorescent cells detected by flow cytometry corresponded to HAC loss (Supplementary Figure S5). Quantitative analysis of metaphase chromosome spreads using a HAC-specific probe (see MATERIALS AND METHODS) correlated with the data on HAC loss determined by FACS (see Supplementary Table S2). Therefore, we conclude that the appearance of green fluorescent cells is caused by HAC loss.

**Figure 3:**
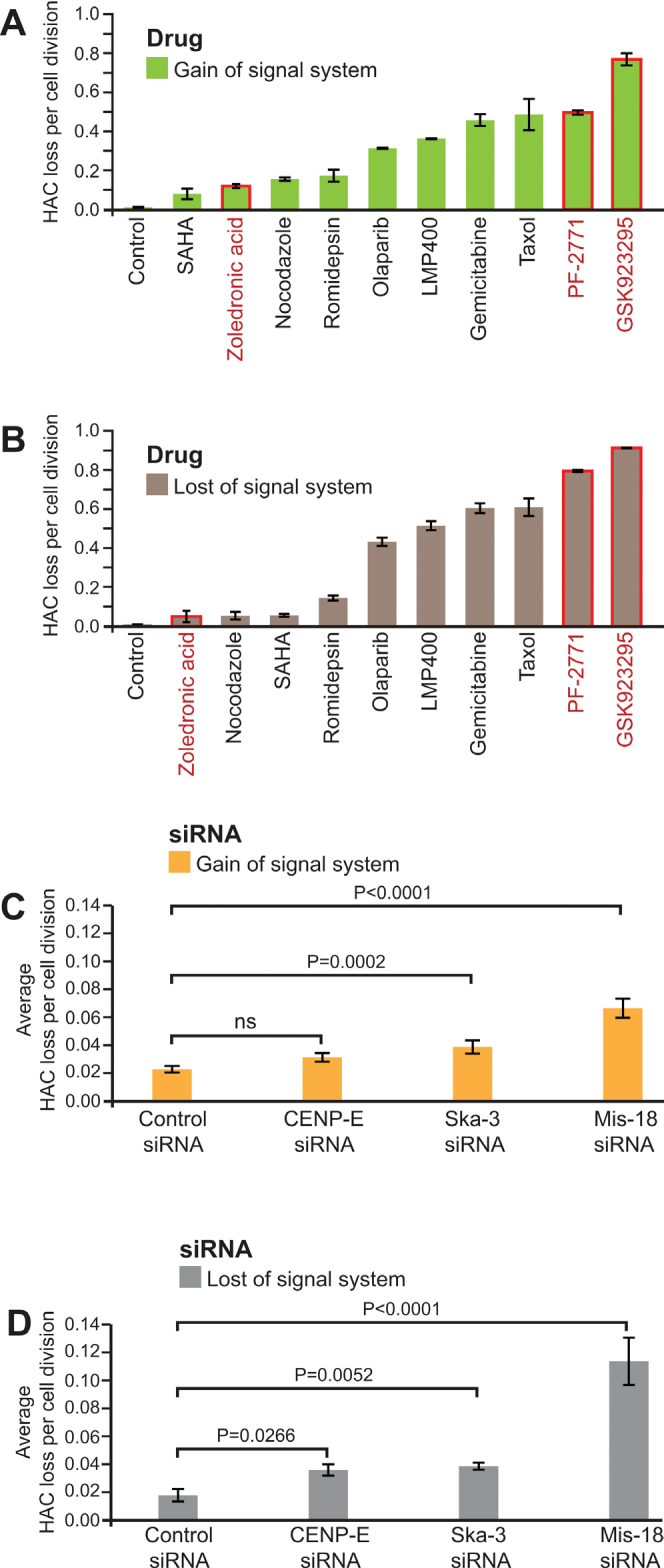
Measurements of CIN induced through treatment by anticancer agents or gene knockouts **A.**, **B.** Effect of anticancer drugs on CIN was measured by “gain of signal” and “loss of signal” HAC-based assays. HT1080 cells containing either the mCherry-shRNA-HAC or EGFP-HAC were treated by ten different compounds as described in MATERIALS AND METHODS. The rate of HAC loss per cell division was calculated based on the ratio of HAC-positive, HAC-negative cells and the average time per cell division. The drug concentrations used were at IC50 for HT1080 cells. The strongest CIN inducers identified were gemcitabine, taxol, PF-2771 and GSK923295. The control corresponds to the frequency of spontaneous HAC loss in HT1080 cells. **C.**, **D.** Effect of gene knockouts on the rate of HAC loss. HT1080 cells carrying either the mCherry-shRNA-HAC or EGFP-HAC were treated by different siRNAs and the rate of HAC loss per generation was calculated as described in MATERIALS AND METHODS. In both systems, the strongest CIN effect was observed after cells treatment with MIS18β siRNA. The control corresponds to a frequency of HAC loss after treatment by a negative control siRNA.

Figure 3A summarizes the estimated rates of HAC loss in response to the analyzed drugs. The rate of HAC loss (R_Drug_) induced by drug treatment was calculated from the proportion of fluorescent cells in the population (see MATERIALS AND METHODS). The data reveal that the drugs affect the rates of HAC mis-segregation during mitotic divisions differently. The highest effect for this group of established drugs was detected for taxol and gemcitabine. A single dose of these drugs increased the rate of HAC loss ∼40 and 50 fold, respectively. To verify the accuracy of measuring HAC loss using this “gain of signal” system, in parallel experiments the effect of the same compounds was measured using the original “loss of signal” system. The results of this analysis are presented in Figure 3B and clearly demonstrate that ranking the drugs according to their effect on HAC loss using two different systems is indistinguishable.

Drugs known to increase CIN beyond the therapeutic threshold are currently few in number, and the clinical promise of targeting the CIN phenotype warrants new screening efforts. Taking this in account, in this work we analyzed three new promising anticancer drugs, PF-2771 and GSK923295 [28] that are inhibitors of the centromere-associated protein CENP-E [29]. (CENP-E is a microtubule plus-end-directed kinetochore motor required for congression of pole-proximal chromosomes [30]) and a potential inhibitor of the kinetochore protein CENP-F, zoledronic acid [31]. Therefore, these inhibitors might induce a high rate of chromosome mis-segregation. Indeed, treatment of cells by PF-2771 and GSK923295 increased the rate of HAC loss to values equal to or exceeding those observed after taxol or gemcitabine treatment (Figure 3A and 3B). Note, these two compounds exhibited the highest effect on HAC loss based on a ranking of 62 anticancer compounds [25].

We conclude that this new “gain of signal” assay is suitable for comparing the effect of various drugs on CIN.

### Quantitative profiling of CIN induced by gene knockouts

One difficulty in identifying mammalian CIN regulators and determining their relative importance in cancer is the lack of a straightforward quantitative assay for CIN in live mammalian cells. To address this point, we analyzed whether the new “gain of signal” system could be applied for detection of CIN induced by the knockout of genes controlling mitotic progression through distinct mechanisms. We performed the experiments on measuring the HAC stability after siRNA depletion of three proteins: the CENP-A loading cofactor MIS18β, the centromere-associated protein CENP-E and a spindle/kinetochore-associated protein SKA3/RAMA1 [29, 32-35]. As seen in Figure 3C, depletion of MIS18β or SKA3/RAMA1 protein significantly increased the frequency of HAC loss. No detectable destabilization of HAC was observed after cell treatment by control siRNA. Control Western blots showed a decrease of corresponding proteins in the treated cells (Supplementary Figure S6). FISH analysis of metaphase chromosome spreads using a HAC-specific probe correlated with the data on HAC loss determined by FACS (Supplementary Table S2). A similar effect of depletion of the same genes was detected using the original “loss of signal” system (Figure 3D). Collectively, the above results validate the ability of both HAC-based assays to be employed for identification of novel genes controlling chromosome transmission in human cells.

### Quantitative profiling of CIN inducing drugs by a fluorescence microplate reader

**Figure 4:**
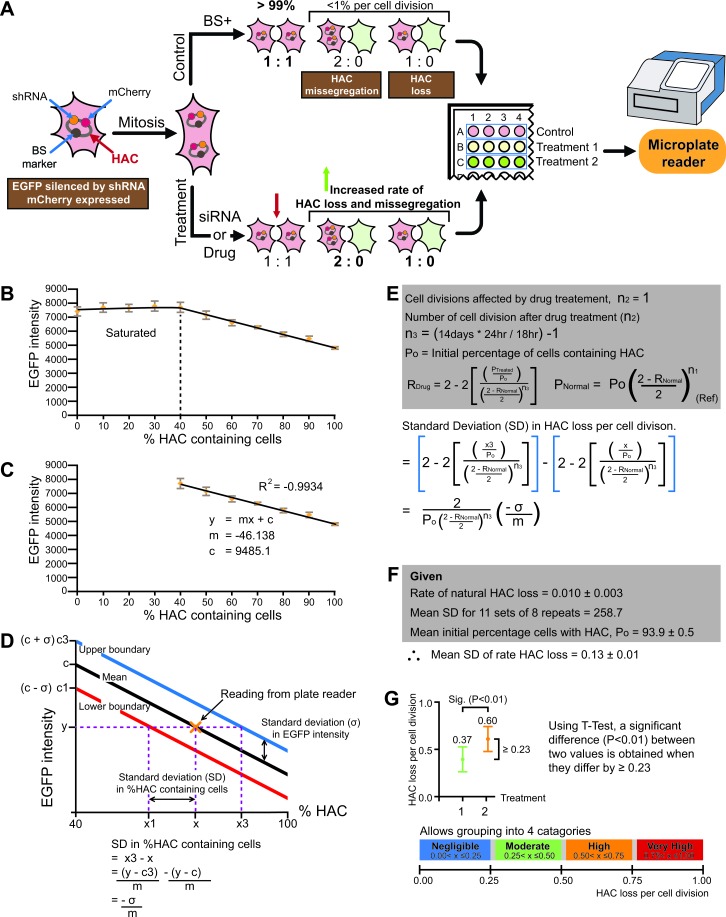
**A.** Scheme of a high throughput assay for measuring chromosome instability (CIN). During S-phase the HAC is duplicated in the cell as other host chromosomes. Under normal growth conditions (control), after cell division the majority of cells in population contain the HAC (1:1 segregation). After drug or siRNA treatment, one copy of HAC can be lost during cell division (1:0 segregation) or two copies of HAC can mis-segregate (2:0 segregation). The cells that inherited the HAC are fluorescent red while cells that lost it are fluorescent green. For each treatment, the average fluorescence of each cell sample may be measured using a multi-well plate reader. Thus, the genes involved in chromosome transmission or drugs affecting chromosome stability may be identified. **B.** An example of a titration curve between EGFP intensity against cell populations of varying percentage of HAC-containing (not green) and HAC-less (green) cells obtained from a fluorescence microtiter plate reader. **C.** Construction of a linear curve between a cell population's EGFP intensity against the percentage of HAC-containing cells. **D.** The relationship between the standard deviation of a cell population's EGFP intensity and it's percentage of HAC-containing cells. **E.** The relationship between the standard deviation of HAC-containing cells and the ‘rate of HAC loss per cell division'. **F.** The estimated standard deviation in the ‘rate of HAC loss' using data derived from a fluorescence plate reader and known rates of natural HAC loss. G. The minimum difference between two values of ‘HAC loss' needed to be significant (*P* < 0.01) was calculated using a T-test and the standard deviations derived above. The results indicate that plate reader is only able to discriminate drugs into broad categories.

In order to efficiently search chemical or siRNA libraries for drugs or gene knockouts inducing CIN, it is necessary to develop a high-throughput screen (Figure 4A). To that end, we performed a set of pilot experiments. First, we demonstrated that it is possible to construct a titter curve using microplate reader data of EGFP fluorescence from populations of known composition of HAC-containing (not green) and HAC-less (green) cells. A titter curve was generated by mixing two cell populations, the HT1080-shGFP-4 cell line which contains the HAC (not green), and the HT1080-JH1 expressing EGFP and without HAC (green) in varying compositions ranging from 0% to 100%. The green fluorescence of these mixed populations was determined with a microplate reader (Figure 4B and 4C). Using the standard deviation of the plate reader data, we found that a significant difference at (*P* < 0.01) could be detected if the rate of HAC loss per cell division of two drug treatments differed by 0.23 (Figure 4D-4F) (see for details MATERIAL AND METHODS). This resolution is insufficient to rank drugs against one another. However, it is sufficient to grade candidate CIN drugs into 4 broad categories (Figure 4G). The standard deviation in the calculated rate of HAC loss was found to be proportional to $\frac{\sigma }{m}$. Thus, better resolution between candidate drugs can be obtained by either decreasing the standard deviation (σ) of the microplate readings or by increasing the difference in green fluorescence intensity between HAC-less cells and HAC-containing cells (m).

**Figure 5:**
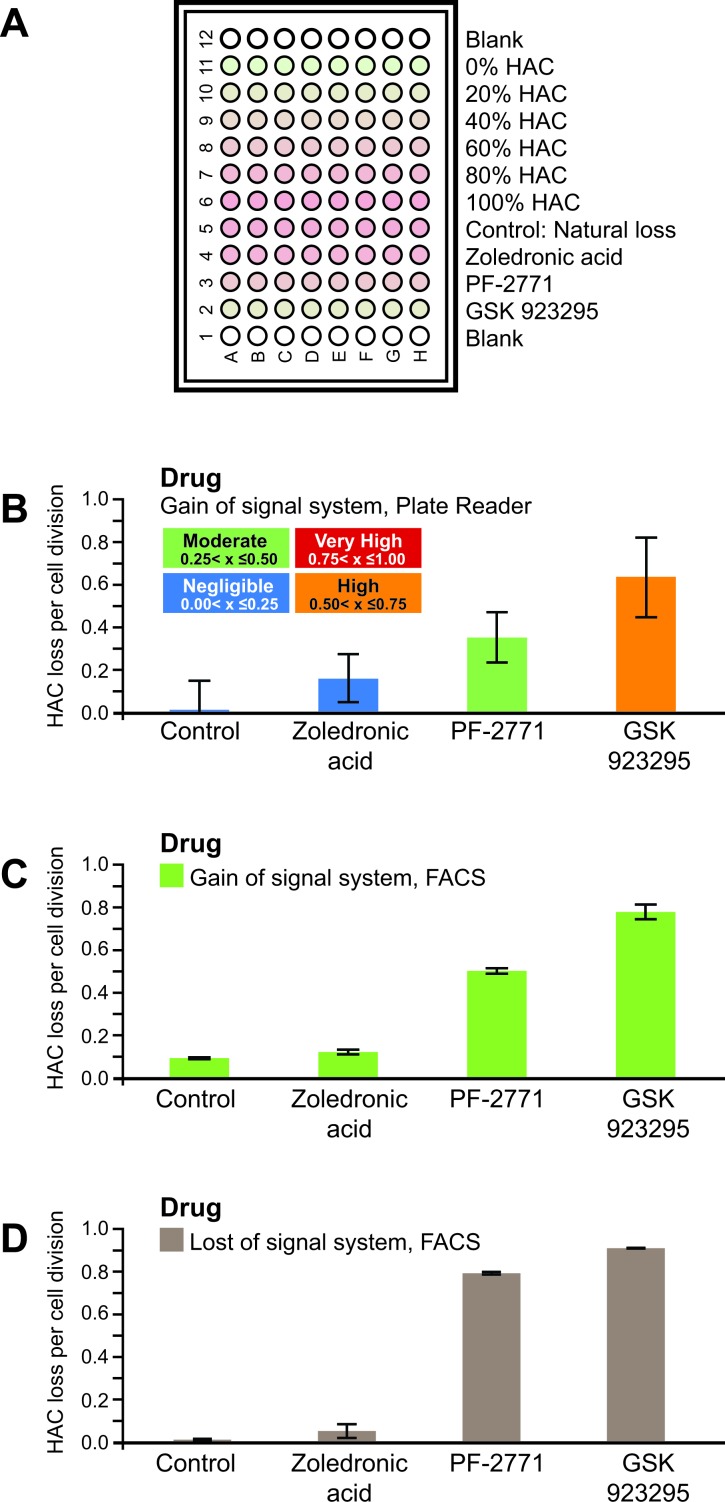
**A.** An illustration of how a 96-well plate was loaded with drug treated cells. Populations of known mixtures of HT1080-shGFP-4 (HAC+) and HT1080-JH1 (HAC-) cells were included as a ladder. **B.** The calculated rates of HAC loss per cell division for three drugs, zoledronic acid, PF-2771 and GSK923295, using the “gain of signal” system with a fluorescence microtitter plate reader. **C.** The calculated rates of HAC loss for the same drugs using the “gain of signal” system with FACS. **D.** The calculated rates of HAC loss for the same drugs using the ”loss of signal' system with FACS.

Then we analyzed three anticancer drugs, PF-2771, GSK923295 and zoledronic acid, using a microplate reader. As seen from Figure 5, treatment of HAC-containing cells by PF-2771 or GSK923295 experienced a dramatic increase in the rates of HAC loss (Figure 5C and 5D) that could be quantified by a fluorescence microplate reader (Figure 5B). Thus, the “gain of signal assay” may be adapted to a 96-well microplate format and used to detect the fluorescence after treatment by a compound.

## DISCUSSION

Most solid tumors are aneuploid, carrying an abnormal number of chromosomes, and they frequently mis-segregate whole chromosomes in a phenomenon termed chromosome instability (CIN). Despite its importance in cancer etiology and therapy, until recently there were no fast, straightforward, quantitative assays for CIN. To address this problem, previously we developed quantitative CIN assay based on a Human Artificial Chromosome (HAC) carrying a constitutively expressed *EGFP* transgene [15]. In this assay, HAC loss events induced by drug treatment could be measured as the fraction of cells that lose EGFP fluorescence signal using routine flow cytometry. This “loss of signal” assay is simple and reproducible and it was successfully used to rank a set of different compounds for their effect on CIN [25]. Unfortunately, this assay is not suitable for the development of a high-throughput screening method. It is difficult to detect the loss of EGFP fluorescence in a few cells against a bright background of EGFP-positive cells. However the opposite, the gain of EGFP fluorescence against a background of colorless cells is much easier to detect. Thus, an assay based on “gain of signal” would potentially be more suitable for high-throughput screening and perhaps earlier detection of the induced CIN phenotype.

In this report, we have described the development and validation of a novel quantitative HAC-based assay for assessing chromosome stability in human cells. A key design feature of this assay is that the fluorescence signal is gained in the cells after loss of a HAC that carries a constitutively expressed shRNA against an *EGFP* transgene integrated elsewhere into the human genome. Thus, cells that inherit the HAC do not display green fluorescence, while cells lacking the HAC do. To verify the accuracy of this new assay, in parallel experiments we measured the rates of HAC loss in a response to cells treatment by a set of anticancer drugs using “gain of signal” and “loss of signal” systems with flow cytometry as a read-out. The relative ranking of drugs that strongly induced CIN did not change, although the same could not be said for drugs that weakly induced CIN. As the aims of this system was to identify drugs that strongly induced CIN, the new “gain of signal” assay may be used both for ranking drugs on their effect on CIN and also for identifying new compounds that promote CIN. During these experiments we identified two drugs, i.e. the newly developed inhibitors of the centromere-associated protein CENP-E, PF-2771 and GSK923295, which induce an extremely high rate of HAC loss. These compounds along with several other drugs that greatly increase CIN, including paclitaxel, olaparib, talazoparib, LMP400 and gemcitabine [25], may be considered as a first choice when CIN is considered as a target for cancer therapy.

In addition, we demonstrated that both HAC-based systems, i.e. “loss of signal” and “gain of signal”, are sensitive enough for detection of CIN induced by depletion of genes, for example, controlling mitotic progression through distinct mechanisms. We demonstrated that siRNA depletion of two genes, *MIS18*β and *SKA3*/*RAMA1*, was accompanied by HAC destabilization to a greater or lesser extent. At present, approximately 400 human genes which control transmission of chromosomes have been annotated with gene ontology (GO) terms, while systematic *CIN* gene screens in yeast have revealed 692 genes [5]. Therefore, in the future these HAC-based assays may be useful in genome-wide screening to uncover novel *CIN* genes in humans. The identification of a comprehensive set of genes controlling chromosome segregation should expedite the development of new therapeutic strategies to target the *CIN* phenotype of cancer cells.

We also examined the potential of the HAC-based “gain of signal” assay in a high-throughput screen of chemical libraries and identification of new genes that modulate CIN (e.g. using multiple gene depletion screens). The pilot experiments were performed where we combined the assay with a 96-well fluorescence microplate reader (see Figures 4 and 5). Although the fluorescence microplate reader was far less sensitive than the flow cytometer, we were able to broadly quantify CIN inducing drugs into 4 categories (Negligible, Moderate, High and Very High) and quantitatively distinguish the drugs by a fluorescence microplate reader.

It is worth noting that this method has some limitations due to the fluorescence signals detection. We expect to improve the present assay by: i) increasing the expression of the *EGFP* transgene by generating new HAC-host cell lines with *EGFP* integrated into different chromosomal sites; ii) expressing multiple shRNA targeted to different parts of the *EGFP* mRNA and, thus, improving silencing; iii) reengineering the mCherry to be constitutively expressed and act as a marker for cell density. In addition, the sensitivity of the assay may be significantly improved using another plate reader system for detection of a fluorescence signal. This work is currently in progress. Also, the current labeling of the HAC does not allow us to simply distinguish between HAC loss (1:0) and HAC mis-segregation (2:0) events. (It is possible only by using a time-consuming interphase FISH). In principle, this limitation may be overcome by expression of the tetracycline repressor fused to mCherry (TetR-mCherry). In such cells the alphoid^tetO^-HAC may be monitored the live following TetR-mCherry signal. Alternative may be loading a copy number dependent, selectable marker (e.g. the dihydrofolate reductase gene) into the HAC where an increased copy number of this gene is accompanied by a higher resistance of cells to methotrexate.

To summarize, a novel “gain of signal” HAC-based assay allows straightforward, quantitative assessment of CIN under a variety of conditions. The assay will be employed to identify novel agents that specifically elevate chromosome mis-segregation and drive lethal aneuploidy. The assay is also suitable for synthetic lethal screens in which combinatorial effects of disruption of two or more pathways can be studied. In future, this “gain of signal” assay may be adapted for high-throughput chemical screens using a fluorescence microplate reader to characterize chemical libraries and identify new conditions that modulate CIN levels, thereby laying the foundation for new treatment strategies for cancer.

## MATERIALS AND METHODS

### Cell lines and culture

Human fibrosarcoma HT1080 cells were cultured in Dulbecco's modified Eagle's medium (DMEM) (Invitrogen) supplemented with 10% (v/v) tet system-approved fetal bovine serum (Clontech Laboratories, Inc.) at 37^°^C in 5% CO_2_. Hypoxanthine phosphoribosyltransferase (HPRT)-deficient Chinese hamster ovary (CHO) cells (JCRB0218) carrying the alphoid^tetO^-HAC [22, 24, 25] were maintained in Ham's F-12 nutrient mixture (Invitrogen) plus 10% FBS with 8 μg/ml of BS (Funakoshi). After loading of the mCherry-shRNA cassette (see below) into the alphoid^tetO^-HAC, the CHO cells were cultured in 1× HAT supplemented medium.

### Construction of the tDNA- mCherry- shRNA plasmid (A245)

Scheme of construction of the A245 plasmid is presented in Supplementary Figure S2. This plasmid was constructed in three steps. In step 1, the HuSH pGFP-V-RS plasmid (OriGene) was digested with BamHI /HindIII and the shGFP sequence was ligated in as a double stranded oligo formed by annealing primers J001 and J002. The plasmid created was HuSH-pRS-shGFP. In step 2, the pcDNA 3.1(+) (Thermofisher Scientific) was digested with HindIII and XbaI. Then the mCherry fragment was PCR amplified from the pRSET-B-mCherry plasmid (Tsien Lab, University of California, San Diego) using the primers J005 and J006, digested with HindIII and XbaI. The two fragments were ligated, creating the plasmid pcDNA-mCherry. In step 3, the A245 plasmid was completed in a 3-way ligation of the following fragments: the U6-shGFP fragment, obtained by PCR amplification of HuSH-pRS-shGFP plasmid using the primers J003 and J004, followed by digestion with SpeI and XhoI; the tDNA-loxP-3′HPRT fragment, obtained by SpeI / BamHI digest of the CAG-EGFP-tDNA plasmid [26]; and lastly the CMV-mCherry-BGHpA fragment which was obtained by PCR amplification of pcDNA-mCherry using the primers J007 and J008, followed by digestion with XhoI and BamHI. Sequences of all primers used are in Supplementary Table S1.

### Construction of the tDNA-EGFP-HyTK plasmid (A158)

Scheme of construction of the plasmid A158 is presented in Supplementary Figure S4. The plasmid A158 was constructed in three steps. In step 1, the plasmid A154 was constructed in a 3-way ligation of three fragments: the tDNA-CAG fragment, obtained from EcoRI/SacI digestion of the CAG-EGFP-tDNA plasmid [26]; the pBS backbone, obtained from SacI / BamHI digestion of pBlueScript II KS (Stratagene); and the EGFP fragment, obtained by PCR amplification of the plasmid p264 [22] using the primers B119/B052, followed by BamHI/EcoRI digestion. In step 2, the plasmid A156 was constructed in a 3-way ligation of three fragments: the T2A-HyTK fragment, obtained from BamHI /XhoI digestion of the CAG-attB-HyTK plasmid [36]; the tDNA-P fragment, obtained by PCR amplification of pCR4-TOPO-tDNA [27] using the primers B405 / B404 followed by SalI / NotI digestion; and the pBS backbone, obtained by NotI /BamHI digestion of pBlueScript II KS (Stratagene). In step3, the A158 plasmid was completed in a 3-way ligation of the following three fragments: the tDNA-CAG-EGFP fragment was obtained by SacI /BamHI digestion of the plasmid A154; the T2A-HyTK-tDNA fragment was obtained by BamHI / NotI digestion of the A156 plasmid; and the pBS backbone was obtained by PCR amplification of pBlueScript II KS (Stratagene) using the primers B396/B395 followed by digestion with NotI / SacI. Sequences of all primers used are in Supplementary Table S1.

### Loading of the tDNA-mCherry-shRNA plasmid into the loxP site of alphoid^tetO^-HAC in hamster CHO cells

3 μg of the A245 plasmid and 1 μg of the Cre expression pCpG-iCre vector DNA were co-transformed into HPRT-deficient hamster CHO cells containing the alphoid^tetO^-HAC by lipofection with FuGENEHD transfection reagent (Roche) or Lipofectamine 2000 (Invitrogen). HPRT-positive colonies were selected after 2 to 3 weeks growth in HAT medium. For each experiment, from 5 to 7 clones were usually selected. Correct loading of the A245 plasmid into the HAC was confirmed by genomic PCR [26] with a specific pair of primers that diagnose reconstitution of the *HPRT* gene (Supplementary Table S1).

### Generation of the EGFP-expressing human cell line

The *EGFP*-containing plasmid A158 was linearized with ScaI and transfected into HT1080 cells. Transfectants were selected on DMEM plus FBS medium with 80 μg /ml Hygromycin B (Invitrogen, USA). Of the colonies obtained, the clone HT1080-JH1 exhibited the strongest expression of EGFP was chosen for further experiments to host the HAC that constitutively expressed shRNA against *EGFP*.

### Microcell-mediated chromosome transfer

The alphoid^tetO^-HAC containing the mCherry-shRNA cassette was transferred from hamster CHO cells to the human HT1080-JH1 cell line using a standard microcell-mediated chromosome transfer (MMCT) protocol [22, 37]. 12 μg/ml blasticidin (BS) was used to select resistant colonies containing the HAC. Typically, three to ten BS^R^ colonies are obtained in one MMCT experiment. BS^R^ colonies were analyzed by FISH for the presence of the autonomous form of the HAC. The potential co-transfer of CHO chromosomes was excluded using a sensitive PCR test for rodent-specific *SINE* elements (Supplementary Table S1). The clone HT1080-shGFP-4 was selected for further work and was maintained under selection with 12 μg/ml blasticidin and 80 μg/ml hygromycin B.

### Flow cytometry

Analysis of EGFP expression was performed using LSRFortessa (BD Biosciences) flow cyctometer and BD FACSDiva software [15]. The cells were harvested by trypsin-treatment. Intensities of fluorescence were determined by flow cytometry. 1.0 × 10^4^ cells were analyzed for each cell sample.

### Drug treatment

Ten different drugs were used in our experiments (Table 1). The experiment protocol was as follows. HT1080 cells containing EGFP-HAC were maintained on 6 μg/ml blasticidin selection while HT1080 cells containing mCherry-shRNA-HAC was maintained on 12 μg/ml blasticidin and 80 μg/ml hygromycin B. Approximately 1 × 10^5^ cells were cultured either in the presence or absence of blasticidin selection in parallel with a third culture that was exposed to the agent under examination to test its effect on HAC segregation. The drug concentration applied was adjusted to the IC50 level for each compound which was determined using a proliferation assay described below. Concentrations of drugs and lengths of treatment are presented in Table 1. Subsequently, the drug was removed by performing three consecutive medium washes and the cells were subsequently grown without antibiotic selection for 13 days. At the end of the experiment, cells were collected and analyzed by flow cytometry to detect the proportion of cells that gain or loss EGFP fluorescence. This served as a measure of HAC stability following drug treatment. For all drugs, experiments were carried out in triplicate.

### Treatment by siRNAs

*SKA3/RAMA1*, *MIS18β* and *CENP-E* were depleted using established siRNAs targeting published sequences [29, 32-34]. siRNAs used to target *SKA3*, *CENP-E* and *MIS18β* are listed in Table 1. siRNAs were purchased from Dharmacon (Lafayette, CO). For siRNA treatment, 1×10^5^ / well cells were seeded in 6 well plates before a day of the experiment. Cells were transfected with each siRNAs using lipofectamine-RNAiMAX (Invitrogen). Cells were grown without blasticidine S/hygromycin B selection for 14 days. Silencing efficiency of each protein was monitored by Western blot analysis. On Day 14, cells were collected and analyzed by flow cytometry to detect the proportion of cells that reactivated EGFP fluorescence or lost EGFP signal. All experiments were carried out in triplicate.

### Calculation of the drug induced rate of HAC loss

To calculate the rate of HAC loss per cell division after cell treatment by a single dose of drug, we used the equations;

Eq1 $$P_{\text{Normal}}=P_{0}{\left(\frac{2-R_{\text{Normal}}}{2}\right)}^{n_{1}}$$

[15]

Eq2 $$P_{\text{Treated}}=P_{0}{\left(\frac{2-R_{\text{Drug}}}{2}\right)}^{n_{2}}{\left(\frac{2-R_{\text{Normal}}}{2}\right)}^{n_{s}}$$

[15]

R_Normal_ is the natural rate of HAC loss per cell division. R_Drug_ is the drug induced rate of HAC loss per cell division. P_0_ is the percentage of EGFP(+) cells when cultured under HAC selection. P_Normal_ is the percentage EGFP(+) cells when cells are cultured without selection. n_1_ = the number of cell doublings that occurred during culturing without selection. P_Treated_ is the percentage of EGFP(+) cells at the end of the experiments. n_1_ is the number of cell doublings that occurs during drug treatment. As drug treatment was limited to less than 18 hrs, drug induced HAC loss was limited to a single cell cycle, n_1_ = 1. n_2_ is the number of cell doublings that occurs during the culturing without selection after the drug treatment. The doubling time of HT1080 is approximately 18 hrs.

### Calculation of the siRNA induced rate of HAC loss

We were unable to quantify the number of cell divisions that siRNA knockdown impacted. Hence, we chose to calculate the average rate of HAC loss per cell division for entire duration of the siRNA experiment. The equation used was $P_{N}=P_{0}{\left(\frac{2-R_{\text{Lost}}}{2}\right)}^{n_{4}}$. P_N_ is the percentage of EGFP(+) cells at the end of the experiments. n_4_ is the number of cell doublings that occurs during the experiment. We estimated this value by counting the size of the cell population at three time points. The first time point was on day the wells were seeded. The second time point was on Day 3, 48 hrs after siRNA transformation. After which, 10% of these cells were plated onto a 75cm^3^ cell culture flask and left to grow until Day 14. The third time point was on Day 14. Cell counts were made using a hemocytometer and viability was determined by 5 min staining with DRAQ7 (Adcam) at concentration stated by the manufacturer and flow cytometry.

### Cell viability test

MTS tetrazolium cell viability assays were done according to the manufacturer's instructions (CellTiter 96 AQueous Assay reagent; Promega). Briefly, the CellTiter 96 AQueous One Solution Reagent was added to each well and incubated at 37°C for 3 hr. Cell proliferation was determined by measuring the absorbance at 490 nm using a microplate reader (Molecular Devices). The half-maximal inhibitory concentration (IC50) was obtained from the MTS viability curves using GraphPad Prism 5. Experiments were carried out in triplicate.

### FISH analysis with the BAC probe

HT1080 cells were processed for fluorescence in situ hybridization (FISH) after drug treatment followed by the 14 day washout. The probe used for FISH was BAC32-2-mer(tetO) DNA containing 40 kb of alphoid-tetO array cloned into a BAC vector as described previously [16]. Specifically, a BAC32-2-mer(tetO) clone contains an amplified synthetic alphoid DNA dimer. One monomer of this dimer is an alphoid DNA consensus sequence carrying the tetO sequence; another monomer is alphoid DNA from chromosome 17. This probe is specific to the HAC but also gives a low signal with centromeric regions of several endogenous chromosomes. BAC DNA was digoxigenin-labeled using a nick-translation kit with digoxigenin-11dUTP or biotin16-dUTP (Roche Diagnostics). Images were captured as before. The probe was denatured for 5 min at 95°C and added to the slides, which were incubated at 72°C for 2 min before overnight incubation at 39°C. After washes with 0.1 × SSC at 65°C followed by a wash with 4 × SSC + 0.1% Tween 20 at room temperature, standard procedures were used to detect biotinylated probes. Slides were mounted with VectaShield and screened for the presence or absence of the HAC. Between 70-150 metaphases were analyzed for each drug treatment.

### Genomic DNA preparation and PCR analysis

Genomic DNA for PCR analysis was prepared using a QIAmp DNA Mini Kit (QIAGEN). Reconstitution of the *HPRT* gene after Cre/lox-mediated recombination was determined by specific primers Lox137-R, Rev#6 and SV40 PA term rev (Supplementary Table S1). Cross contamination by hamster chromosomes after HAC MMCT transfer from hamster cells to human cells was determined by specific primers detecting hamster *SINE*s: Cons B2-F, Ham B2-F and Ham B2-R (Supplementary Table S1). PCR products for sequencing were separated by agarose gel electrophoresis and gel extracted.

### Western blot analysis

siRNA depletion of proteins was confirmed by Western blot. The following antibodies were used for immunoblotting: Anti-OIP5 (MIS18β) antibody (ab168516), Anti-SKA3 antibody (ab175951), Anti-GAPDH antibody (Cell Signaling).

### Fluorescence microplate reader

20,000 cells / well were plated onto a 96 well, treated, flat bottom, tissue culture imaging plate, (BD falcon, REF 353219) and left to grow overnight with 80 μg/ml hygromycin B. On the next day, complete confluence was achieved, and the cells cultures were washed twice with PBS (Life Technologies) with 0.9 mM CaCl_2_ and 0.5 mM MgCl_2_. Cells were then lysed with 50 μl of CelLytic Sigma (C2978-50ml) and immediately read with a fluorescence microplate reader (Synerg HT Biotech) (Figure 4A)

### Calculation of the rate of HAC loss using a microplate reader

Two cell populations, the HT1080-shGFP-4 cell line which contains the HAC (not green) and the HT1080-JH1 expressing EGFP and without HAC (green) were mixed in varying compositions ranging from 0% to 100%. A titter curve was generated. The green fluorescence of these mixed populations was determined with a microplate reader (Figure 4B). The experiment was repeated three times, each with 8 replicates. The titration generated a linear curve between EGFP intensity and cell populations that ranged from 40% to 100% HAC (R^2^ = −0.9934) (Figure 4C). Below 40% HAC-containing cells in population, the plate reader was saturated at the settings used.

The average standard deviation from microplate reader readings was determined and used to generate two parallel lines, one standard deviation above and below the mean linear curve. As illustrated in Figure 4D, these two curves were used to determine the standard deviation in the percentage of green fluorescence cells. Then equations Eq1 and Eq2 were used to derive the standard deviation of the rate of HAC loss per cell division (Figure 4F). T-test was then used to determined how large a difference between two values is required to obtain a significant difference at *P* < 0.01 (GraphPad Prism 5) (Figure 4G).

In addition, by plugging in the known rate of natural HAC loss in HT1080, the starting percentage of HAC containing cells and all possible value of %HAC in a population into Eq1 and Eq2, we determined that the lowest percentage of HAC-containing cells for a single drug dose was 42%. This is within the detection limit of plate reader at the settings used.

## SUPPLEMENTARY MATERIAL FIGURES AND TABLES


