# Supplementary material for: Development of a novel HAC-based “gain of signal” quantitative assay for measuring chromosome instability (CIN) in cancer cells
Source: Oncotarget. 2016 Mar 2;7(12):14841–56. doi: 10.18632/oncotarget.7854 (PMC4924756; doi:10.18632/oncotarget.7854)
Supplement: Supplementary file 1 [file oncotarget-07-14841-s001.pdf]

Development of a novel HAC-based "gain of signal" quantitative assay for measuring chromosome instability (CIN) in cancer cells

Supplementary Material

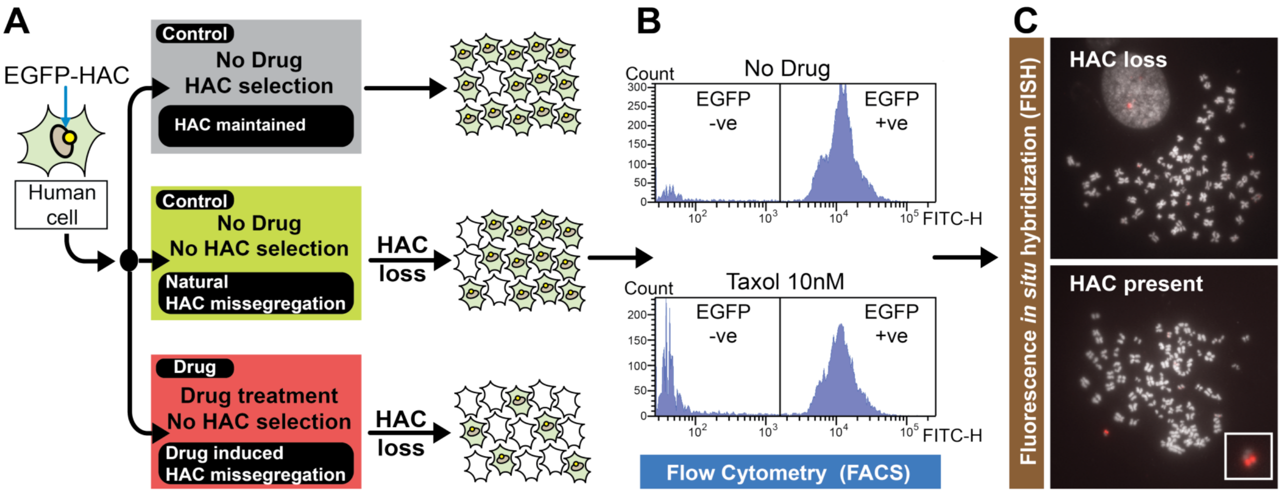

Supplementary Figure S1: Scheme of the original "loss of signal " assay for measuring chromosome instability (CIN) based on the use of HAC containing the EGFP transgene. (**A**) Cells that inherit the HAC display green fluorescence, while cells that lack it do not. The control untreated cells show uniform green fluorescence, while those that have lost HAC after drug treatment are highly variable in fluorescence. Therefore, the actual number of cells with the EGFP-HAC can be measured by flow cytometry (FACS). Thus, the compounds, which increase HAC loss and therefore increase a spontaneous chromosome mis-segregation rate may be identified. (**B**) A flow cytometry histogram illustrating mitotic stability of the EGFP-HAC in human HT1080 cells before and after treatment by taxol. The x-axis represents the intensity of the fluorescence, the y-axis the number of cells. (**C**) FISH analysis of the HAC-containing HT1080 clone and the clone that has lost the HAC after drug treatment. The HAC was visualized using BAC32-2-mer(tetO) DNA probe (red).

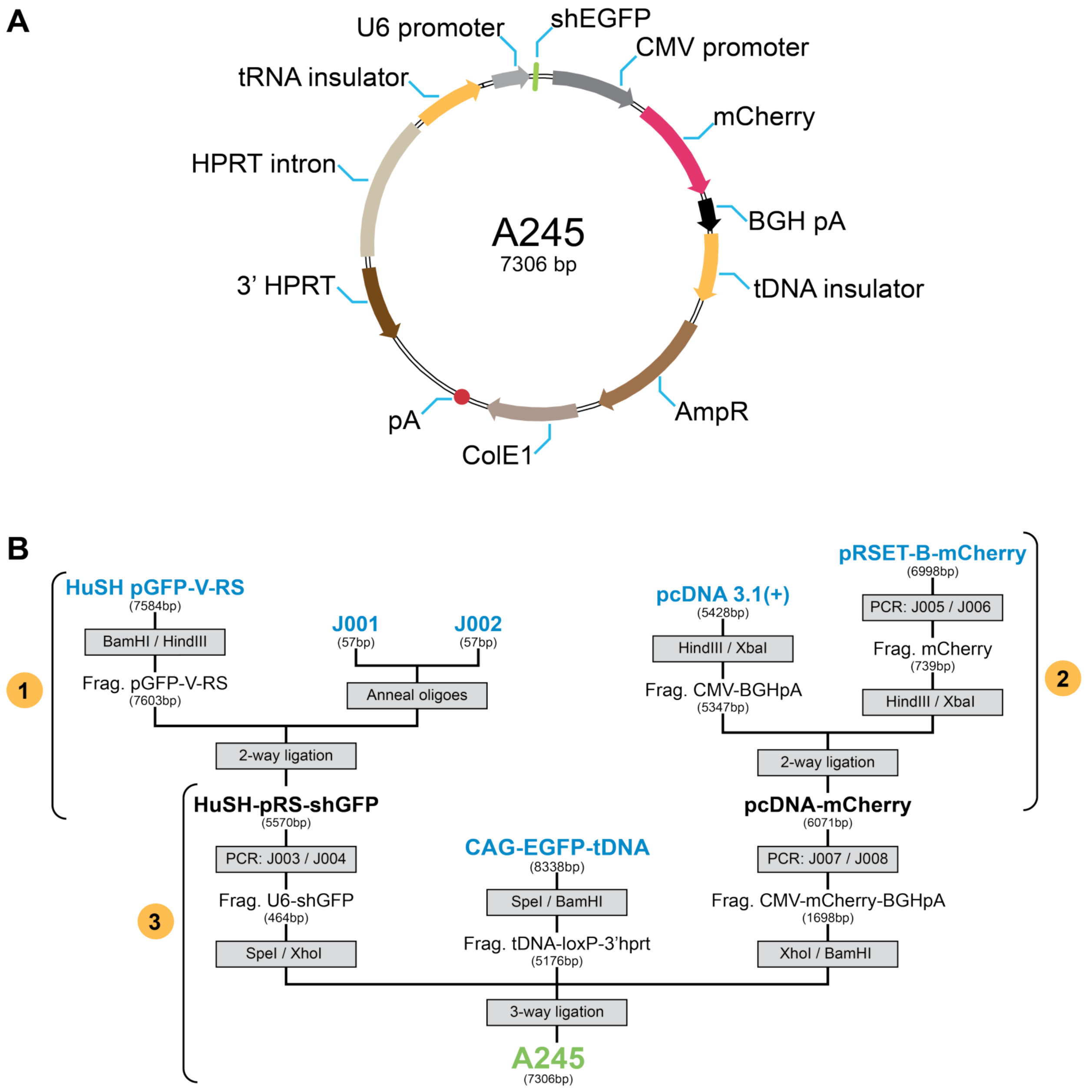

Supplementary Figure S2: Map (**A**) and scheme of construction (**B**) of the A245 plasmid containing the tDNA-shEGFP-mCherry cassette that was inserted into the HAC via Cre-loxP recombination (see details in MATERIALS AND METHODS).

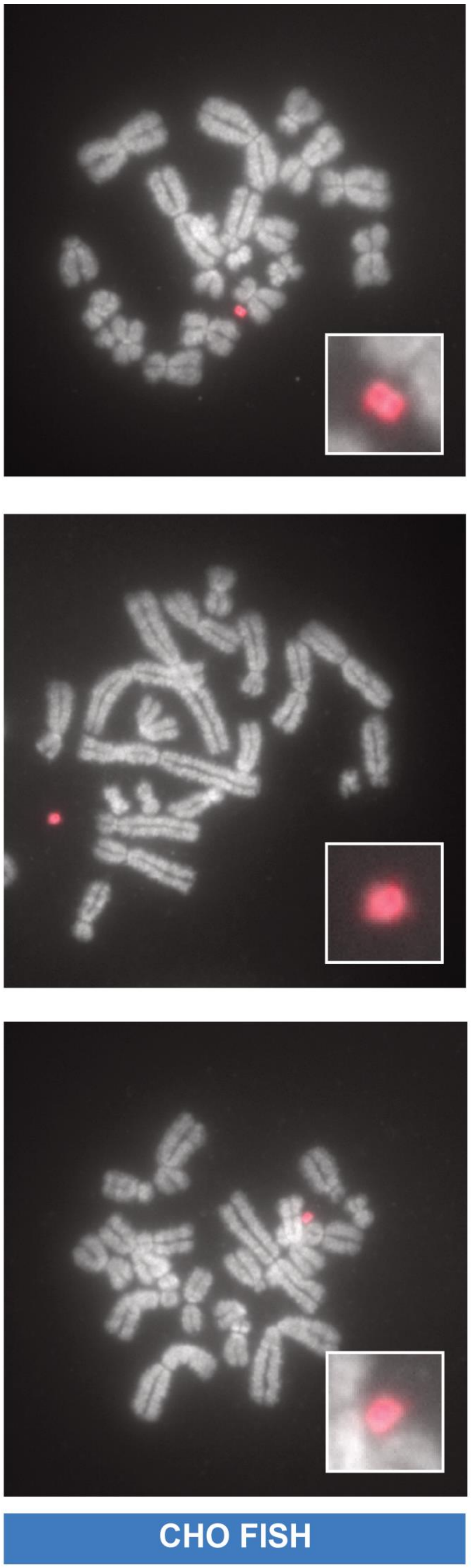

Supplementary Figure S3: FISH analysis of the mCherry-shRNA-HAC in hamster CHO cells using a specific probe for HAC BAC32-2-mer(tetO) (red).

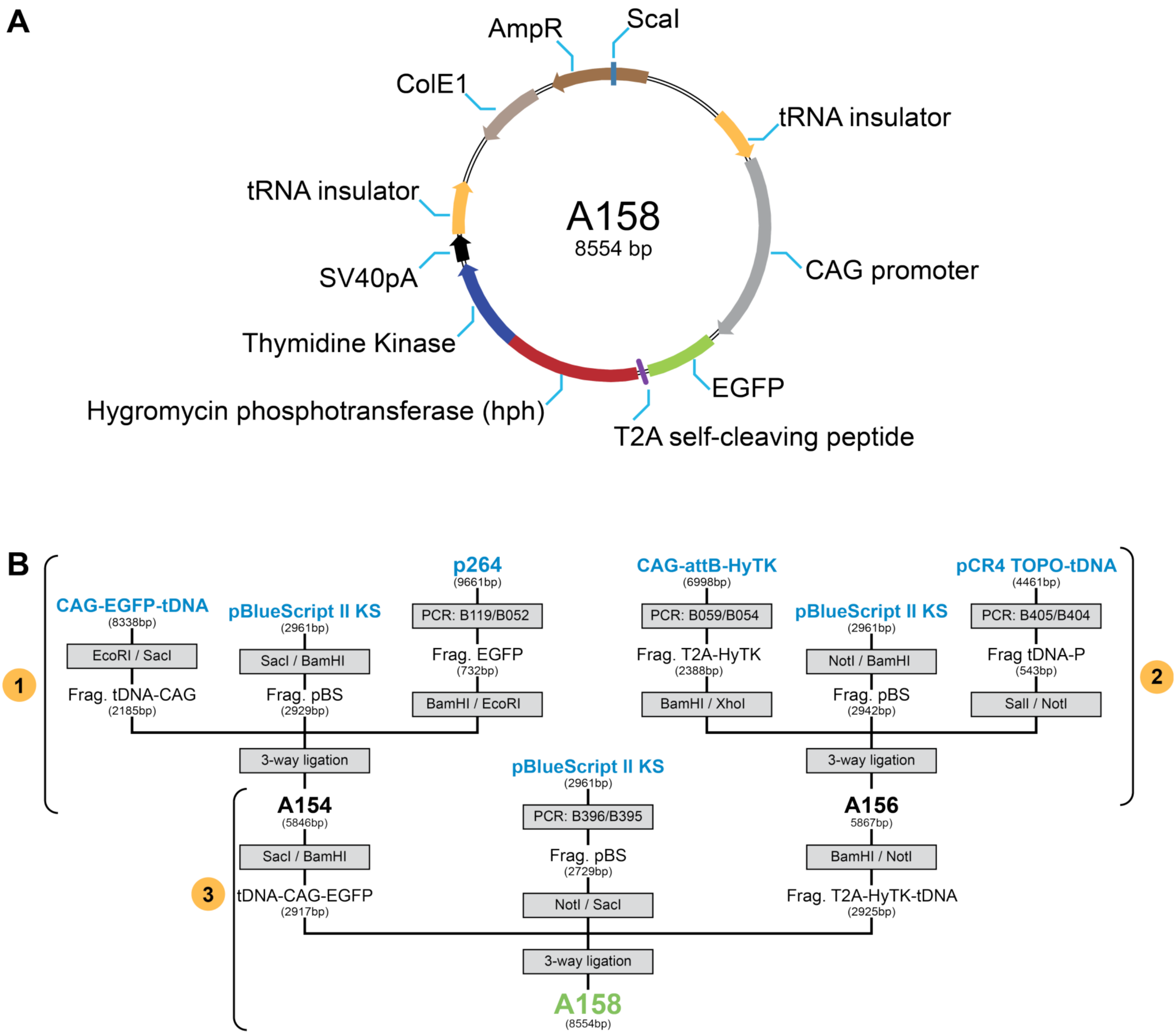

Supplementary Figure S4: Map (**A**) and scheme of construction (**B**) of the A158 plasmid containing the tDNA-EGFP-HyTK cassette and used for insertion into an ectopic chromosomal site in human HT1080 cells (see details in Materials and Methods).

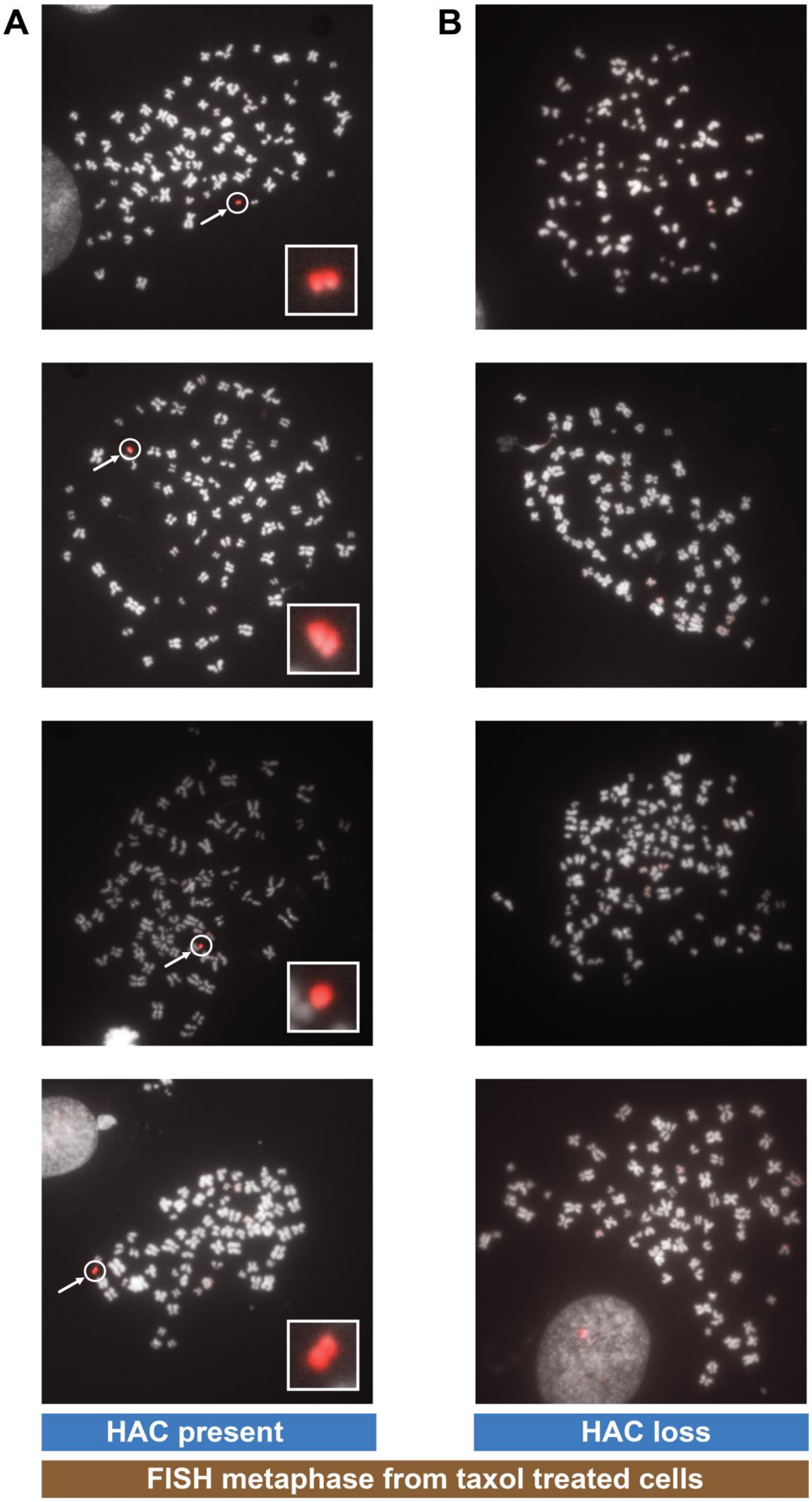

Supplementary Figure S5: Fluorescence *In-situ* Hybridization (FISH) images of human HT1080 cells treated with 10 nM taxol. **(A)** Cells that contain HAC. **(B)** Cells that have lost HAC after taxol treatment. The HAC was visualized using BAC32-2-mer(tetO) DNA probe (red).

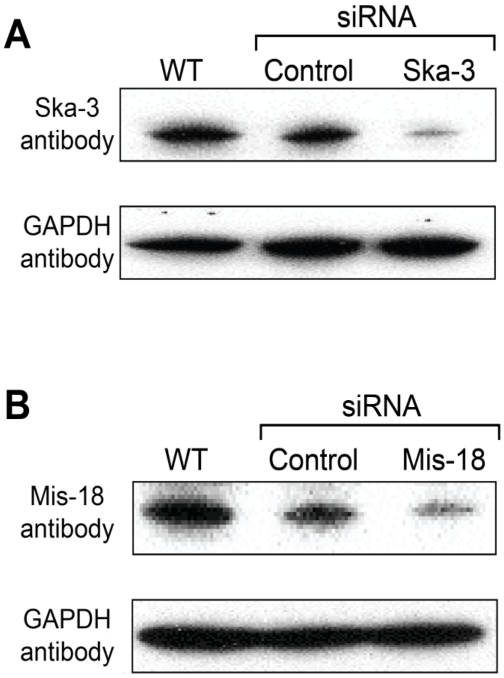

Supplementary Figure S6: Western blot from siRNA knockdown of **(A)** Ska-3 and **(B)** Mis-18 in the "gain of signal" system.

Supplementary Table S1: Primers used in this study

Plasmid A158

ID	Sequence
B052	5′ CGTCACGGATCCCTTGTACAGCTCGTCCATGC 3′
B119	5′ CGAGAGGAATTCGCCGCCACCATGGTGAGCAAG 3′
B059	5′GCTGTGGATCCGGAGAGGGCAGAGGAAGTCTGCTAACATGCGGTGACGTCGAGGAGAATCCTGGCCCAATGAAAAAGCCTGAACTCACCG 3′
B054	5′ CGAGACCTCGAGCCAGACATGATAAGATACATTGATG 3′
B405	5′ CGACCTGTCGACGCGCCAATCCCATTGCAAA 3′
B404	5′CGACAGACTGCGGCCGCGGAGTAGTGCCCCAACTGGGGTAACCTTTGAGTTCTCTCAGTTGGGGGCGTAGTGATGGCTGCATTCCACAC 3′
B396	5′ GCTTCGAGCTCCTAGGTATCAAGATCTGGCGCGCCTTAATTAATCGCCTTGCAGCACATC 3′
B395	5′ CGACTGTGAGCGGCCGCATGGCCGGCCACATACGAGCCGGAAGCATAA 3′

Plasmid A245

ID	Name	Sequence
J001	shGFP F BamH1	5′GATCCAAGCTGACCCTGAAGTTCATTTCAAGAGAATGAACTTCAGGGTCAGCTTGCTTTTTT 3′
J002	shGFP R Hind3	5′AGCTTAAAAAAGCAAGCTGACCCTGAAGTTCATTCTCTTGAAATGAACTTCAGGGTCAGCTTG 3′
J003	U6+shGFPF Spe1	5′ gatcaactagtCAGTGGAAAGACGCGCAGGCAAA 3′
J004	U6+shGFPR Xho1	5′ agtcactcgagCCTGGGGACTTTCCACACCCTAA 3′
J005	mCherry kozacF Hind3	5′ atgctaagcttcgccaccatggtgagcaagggcga 3′
J006	mCherry kozacR Xba1	5′ tagcatctagaTACTTGTACAGCTCGTCCATGCC 3′
J007	pcDNA mCherryF Xho1	5′ atgctctcgagTACGCGTTGACATTGATTATTGAC 3′
J008	pcDNA mCherryR BamH1	5′ tagcaggatccTCAGAAGCCATAGAGCCCACC 3′

HPRT gene reconstitution

Name	Sequence
Lox137-R	5′ AGCCTTCTGTACACATTTCTTCTC 3′
Rev #6	5′ GCTCTACTAAGCAGATGGCCACAGAACTAG 3′
SV40 PA term rev	5′ AATGGTTACAAATAAAGCAATAGCATCAC 3′

Hamster specific B2 repeats

Name	Sequence
Cons B2-F	5′ CCATCTGTAATGAGATCTGATGC 3′
Ham B2-F	5′ GCTCAGAGGTTAAGAGCACTGAC 3′
Ham B2-R	5′ TGCTTCCATGTATATCTGCACAC 3′

Supplementary Table S2: Comparison between FISH and FACS data to evaluate HAC loss induced by drug treatment or siRNA knockdown

**Drug**	**FISH*** % Cells without HAC	**FACS** % Cells with EGFP fluorescence
No selection	20	21.5
Gemcitabine	41	40.2
Taxol	44	37.4
**siRNA**	**FISH** % Cells without HAC	**FACS** % Cells with EGFP fluorescence
No selection	28	23.6
CENP-E siRNA	32	29.1
SKA3 siRNA	37	31.4
MIS18β siRNA	45	40.7

*At least 70-150 metaphases were screened after cells treatment.
